# Physicochemical Properties of Pineapple Stem Fiber/Gellan Gum Biocomposite Films as a Potential Platform for Buccal Drug Delivery

**DOI:** 10.3390/jfb17090468

**Published:** 2026-09-12

**Authors:** Tuty Fareyhynn Mohammed Fitri, Azlin Fazlina Osman, Eid Alosime, Sinar Arzuria Adnan, Nur Hidayah Ahmad Zaidi

**Affiliations:** 1 Faculty of Chemical Engineering & Technology, Universiti Malaysia Perlis (UniMAP), Kompleks Pusat Pengajian Jejawi 2, Arau 02600, Malaysia; tutyfareyhynn@unimap.edu.my (T.F.M.F.); sinar@unimap.edu.my (S.A.A.); hidayah@unimap.edu.my (N.H.A.Z.); 2 Biomedical and Nanotechnology Research Group, Centre of Excellence Geopolymer and Green Technology (CegeoGTech), Universiti Malaysia Perlis (UniMAP), Arau 02600, Malaysia; 3 King Abdulaziz City for Science and Technology (KACST), P.O. Box 6086, Riyadh 11442, Saudi Arabia

**Keywords:** polymer, buccal films, gellan gum, pineapple stem fiber, biocomposite, mucoadhesion

## Abstract

The physicochemical properties of buccal films are vital for evaluating their suitability for mucosal applications. By focusing on these properties, researchers can enhance the mechanical functionality and mucoadhesion of the films. This study aimed to overcome the mechanical limitations of neat gellan gum and to produce biocomposite films with enhanced physicochemical properties and mucoadhesive properties for potential use in buccal drug delivery. Biocomposite films composed of gellan gum (GG) and pineapple stem fiber (PSF), with glycerine as a plasticizer, were prepared using the solvent casting method to develop a formulation suitable for this application. Fourier transform infrared (FTIR) spectroscopy, pH and thickness measurements, tensile test, folding endurance, swelling index, scanning electron microscope (SEM), mucoadhesion test and X-ray diffraction (XRD) analysis were conducted to determine the optimal PSF content in the GG-based biocomposite film formulation. The results indicated that the optimal formulation, GG/3PSF, was achieved with the incorporation of 3 wt% PSF relative to the GG mass. Specifically, the GG/3PSF biocomposite film exhibited a tensile strength of 17.10 ± 0.4 MPa (a 50% increase compared to neat GG), an elongation at break of 46.0 ± 2.5%, a tensile toughness of 41 ± 2.0 MPa, and an ex vivo mucoadhesive residence time of at 7.76 ± 0.51 h for GG/3PSF (compared to 3.90 ± 0.24 h for neat GG). Additionally, it maintained a moderate and optimal swelling index of 115.31 ± 2.3% after 60 min of hydration, which prevents structural instability associated with excessive swelling (such as 161.81 ± 4.7% observed in GG/7PSF), while possessing acceptable thickness (0.09 ± 0.005 mm) and neutral pH (7.0 ± 0.05). The developed buccal film is environmentally friendly due to the utilization of pineapple stem fiber, an agricultural by-product that can reduce material costs compared with synthetic fillers and shows considerable potential as a biocomposite film for buccal drug delivery applications.

## 1. Introduction

Buccal mucosa contact films are gaining increasing importance for local and systemic oral applications due to their painless and comfortable administration, particularly for individuals who experience difficulty swallowing oral solid dosage forms [1]. As the need for innovative polymeric matrices grows, it is crucial to understand the physicochemical and mechanical properties of buccal films. Scientists and researchers continue to explore new natural and synthetic mucoadhesive systems [2,3,4]. They have found that the intersection of material science and pharmacology offers new ways to improve medication administration through buccal channels. Continuous research is advancing the creation of novel materials and drug formulations, thereby facilitating the development of innovative therapeutic strategies. Innovative approaches, meticulous preparation, and an emphasis on patient needs are required due to the problems with the expenses and complexity of the formulation of buccal films [5,6]. The focus of research has been shifted toward developing biocomposite buccal film by utilizing agricultural by-products such as pineapple stem fiber (PSF), which can reduce material costs compared to synthetic fillers, making the production process more economical. Buccal films are generally prepared using polymeric materials that provide the essential characteristics of mucoadhesion, controlled release, and mechanical stability [6,7]. Gellan gum (GG) serves as a suitable material for the production of buccal films [7]. As a natural polysaccharide with inherent gelling properties, it is effective for formulating films designed to dissolve within the oral cavity [7,8,9]. Despite the extensive research conducted on gellan gum (GG) as a film-forming biopolymer, previous investigations have indicated that the integration of cellulose-based and natural fibers can significantly alter the mechanical and structural characteristics of GG films. For instance, studies have reported on gellan gum combined with kenaf core fiber (GG/KCF) biocomposite films, noting that the inclusion of KCF influences the tensile properties, ductility, and stiffness of the resultant films [10]. Furthermore, composite films incorporating gellan gum and cellulose nanofibers have been developed for biomedical purposes, highlighting the ability of cellulose-derived reinforcements to enhance the performance of GG-based films [11]. These investigations illustrate the potential of integrating GG with cellulosic or natural reinforcing phases. However, the application of pineapple stem fiber (PSF), derived from an agricultural by-product, in a low-filler-content GG matrix for the development of buccal films has not been thoroughly explored. Specifically, there is a scarcity of information regarding the correlation between PSF concentration and the cumulative mechanical, swelling, morphological, and ex vivo mucoadhesive properties of GG/PSF films. Consequently, the originality of the current study lies not only in the combination of GG with a natural fiber but also in the assessment of pineapple stem fiber as a sustainable reinforcing phase for GG buccal films. In addition, it aims to identify an optimal PSF concentration that achieves a balance among mechanical strength, flexibility, swelling behavior, and mucoadhesive retention. Gellan gum offers favorable texture, ensures stability, and enables the controlled release of active pharmaceutical substances. Furthermore, its biocompatibility and excellent film-forming abilities are crucial attributes for buccal delivery systems [12]. Gellan gum produces a robust and flexible film that adheres well to the buccal mucosa, thereby improving drug absorption. This material is non-toxic and the body accepts it well, which makes it safe for use in oral-mucosal applications. Gellan gum can be altered so that active pharmaceutical ingredients can be released in a controlled and long-lasting way [7]. It keeps moisture, which aids in increasing the comfort and duration of the film in the mouth [9]. In this research study, gellan gum was mixed with pineapple stem fiber (PSF) as a filler in the formulation of biocomposite buccal films. This combination may improve the qualities of the final product in various ways. The inclusion of PSF can improve the mechanical strength and flexibility of the film, which reduces the chance of breakage and promotes better adherence to mucosal surfaces. The fiber could assist in the controlled release of active ingredients, enhancing their bioavailability and ensuring more effective absorption through the buccal mucosa. Furthermore, both gellan gum and pineapple fiber can decompose naturally, which makes them good for eco-friendly uses [13,14,15]. Pineapple fiber can also provide extra nutritional advantages or functional qualities, like being a source of dietary fiber. It helps to keep moisture, which supports the hydration of the buccal film and stops dryness that might cause discomfort [15,16,17]. The rising interest in natural and health-focused products makes pineapple stem fiber a desirable choice for consumers who care about their health. However, when combining these two elements, it is essential to optimize the formulation to reach the desired qualities according to the specific application and targeted release features. This research aimed on finding the optimal ratio of GG/PSF to innovate and optimize the mechanical properties of the resultant GG/PSF buccal films. This study establishes a robust, eco-friendly biocomposite matrix, which represents a crucial foundational step before conducting active pharmaceutical ingredient (API) loading and in vitro drug release studies in future work.

## 2. Materials and Methods

### 2.1. Materials

Pineapple stem waste was collected from GG Eco Farm (Perlis, Malaysia). Gellan Gum powder (GGP) was purchased from Nichz Ingredient (Selangor, Malaysia). Glycerine was kindly obtained from HmbG Chemicals (Hamburg, Germany). A phosphate buffer solution (PBS) (7.0 pH) was acquired from R&M Chemicals (Selangor, Malaysia). Distilled water was used throughout the process as needed.

### 2.2. Extraction Process of Pineapple Stem Fiber (PSF)

Pineapple plant stems were collected from a pineapple plantation of the MD2 type in Perlis, Malaysia (GG Eco Farm). Dirt and debris were removed from the pineapple stems and the plant stems by washing them thoroughly. Then, the stems were chopped into small pieces to facilitate further processing and extraction of fibers. The chopped stems were soaked in distilled water (1:1) to separate the fibers from woody parts of the stem by natural retting. After retting, the softened stems were ground. This reduced the piece size into paste with crude fibrous material. The fibrous materials were filtered out with filter cloth, and the extracted fibers were rinsed thoroughly to remove any remaining non-fibrous parts and retting residues. The fibrous materials were centrifuged (4000 rpm for 10 min) to optimize the extraction and preparation of fibrous pineapple stem fiber, hence improving their quality, efficiency, and consistency for intended uses. The fibrous materials then underwent a drying process at 60 °C for 48 h. The dried pineapple stem fiber was ground and sieved using a 53-micron sieve and kept at room temperature for further use.

### 2.3. Preparation of GG Biocomposite Buccal Films

GG biocomposite buccal films were prepared with the solvent casting method. In order to produce a piece of rectangular-shaped biocomposite buccal film with a size of 14 cm × 11 cm, accurately weighed quantities of GG (1 g) were dispersed in 30 mL of distilled water. The mixtures were stirred on a magnetic stirrer at 50 rpm for 15 min. Next, PSF (1 wt%, 3 wt%, 5 wt%, and 7 wt% relative to the mass of GG, which are 0.01 g, 0.03 g, 0.05 g and 0.07 g of PSF per 1 g GG, were added into the mixtures and stirred for 15 min. Then, glycerine (1 g) with 20 mL of distilled water was added (as plasticizer), and the stirring continued for 15 min until it reached 75 °C. A quantity of 30 mL of each colloidal dispersion was poured on the flat surface, and dried at room temperature for 48 h, allowing us to obtain thin films after solvent evaporation. The GG biocomposite buccal films were removed from the mold, and the films were cut into the desired shape and dimension before proceeding with the several testing procedures. The composition of the prepared mucoadhesive film formulations is presented in Table 1.

## 3. Testing and Characterization

### 3.1. Attenuated Total Reflectance-Fourier Transform Infrared (ATR-FTIR) Spectroscopy

GGP, PSF, Neat GG and GG biocomposite oral buccal film samples were analyzed with the Perkin Elmer Paragon FTIR (Waltham, MA, USA) spectrometer with Attenuated Total Reflectance (ATR) in the range of 650–4000 cm^−1^ at a 32 cm^−1^ resolution and 32 scans.

### 3.2. pH and Thickness Measurement

The biocomposite buccal film samples, measuring 2 × 2 cm, were immersed in 10 mL of phosphate buffer solution at pH 7.0 for a duration of 3 h. During this period, the samples underwent sonication for 10 min every hour. Subsequently, the solutions were filtered, and the pH levels were measured utilizing a digital pH meter (Hanna Instruments, HI 98103). The thickness of the films was measured at six different locations using a digital micrometer (Mitutoyo digital calipers). Thickness (n = 6) measurements were taken across different spots to ensure spatial uniformity across the cast films. The average thickness was calculated.

### 3.3. Tensile Strength

The mechanical properties of the biocomposite buccal film samples were determined by a standard tensile test. All measurements were carried out at room temperature (23 ± 0.5 °C) for specimens with a constant cross-section cutout from the prepared films. Five replicate specimens (n = 5) were prepared and tested for each formulation according to the dimension stipulated in the ASTM 638 Type V. The Instron machine model-5569 (Norwood, MA, USA) was used to conduct the tensile test under a crosshead speed of 2 mm/min. The tensile test properties such as tensile strength, Young’s modulus, elongation at break and tensile toughness were calculated, and the outcomes were reported as mean ± SD of the measurements.

### 3.4. Folding Endurance Analysis

A small strip measuring 4 cm × 1 cm was folded repeatedly at the same location in a 180° plane until it broke, or a maximum of 300 times without breaking in accordance with ISO 5626 (modified) standards, to quantitatively measure the film’s folding endurance. Folding endurance was measured by counting the number of times a film can be folded without breaking.

### 3.5. Swelling Index

The swelling index was used to assess the swelling behavior of biocomposite buccal films in contact with saliva and determine their suitability for oral cavity use. The swelling index was calculated according to Equation (1) by weighing 1 × 1 cm pieces of mucoadhesive buccal film, before (*X*1) and after immersion in a simulated fluid (*X*2).

(1) $$\%\;\mathrm{Swelling}\;\mathrm{index}=\frac{X2-X1}{X1}\times 100$$

The film samples were placed in a test tube and left to swell in a phosphate buffer solution with a pH of 7.0 in a water bath at 37 ± 1 °C. At predetermined times (15, 30, 45, and 60 min), the swollen films were removed from the swelling medium and weighed again. To remove excess water from their surface, filter paper was used. The swelling test was performed using three replicate specimens (n = 3) for each formulation at each sampling time, and the results were expressed as mean ± SD.

### 3.6. Scanning Electron Microscope (SEM)

The fractured surface morphology of specific samples following tensile failure (Neat GG (control sample), GG/3PSF biocomposite (optimal sample), and GG/7PSF biocomposite (suboptimal sample)) was examined and analyzed via scanning electron microscopy (SEM) using a TESCAN VEGA model. Prior to imaging, all specimens were coated with platinum using a JFC-1600 Auto Fine Coater (JEOL Ltd., Akishima, Japan).

### 3.7. Mucoadhesion Test

**Figure 1:**
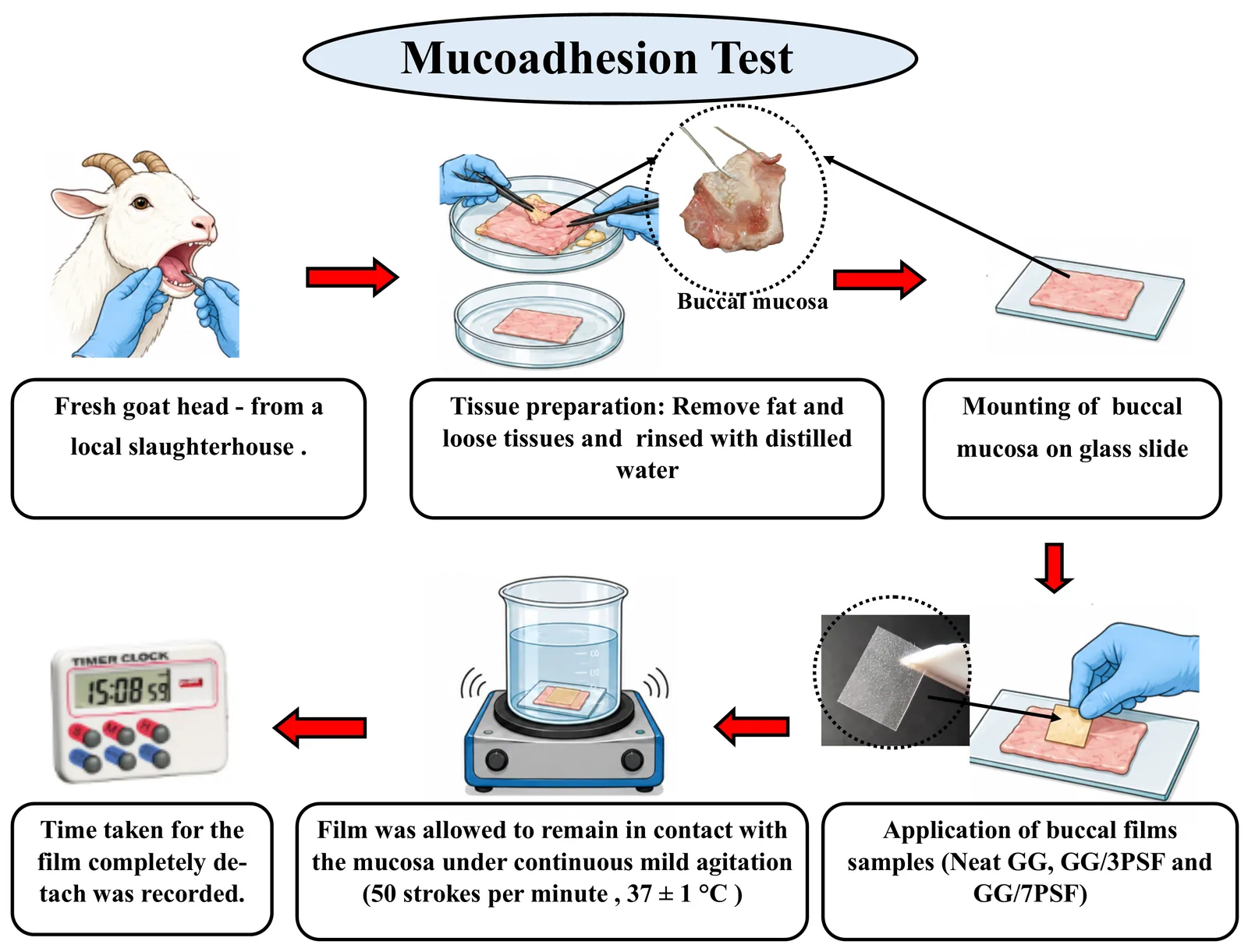
The methodology employed to evaluate the mucoadhesion time of the buccal films.

The oral mucosa of various animal species was subjected to testing; however, pigs’ and goats’ oral tissues were predominantly utilized for ex vivo mucoadhesion studies due to their structural and functional similarities to human oral mucosa. In the present investigation, buccal mucosa from a deceased goat was used to assess mucoadhesion of the buccal film samples. An ex vivo mucoadhesion residence time test was performed according to the method reported by Pawar et al. [18]. To account for inter-animal variability, fresh goat buccal mucosa was obtained from three separate animal specimens (n = 3) at a local slaughterhouse. After carefully removing the underlying fat and loose tissues, the mucosal membrane was washed with distilled water and adhered to a glass slide. The mounted mucosa was placed in a beaker with 150 mL of pH 7.0 phosphate buffer solution, which was kept at 37 ± 1 °C in a shaking water bath. To replicate oral shear pressures, 50 strokes per minute of continuous, mild agitation was used. Light finger pressure was used to apply the chosen buccal film samples (Neat GG, GG/3PSF, and GG/7PSF) (1 cm × 1 cm) to the buccal mucosal surface for 30 s. The amount of time the film stayed affixed to the buccal mucosa until it completely separated was noted. The amount of time needed for the film to completely separate from the mucosal surface was used to assess mucoadhesion. Figure 1 illustrates the overall procedures performed for this ex vivo mucoadhesion test.

### 3.8. X-Ray Diffraction (XRD)

To determine crystallinity, XRD analysis of the films and their components was organized. The samples of GGP, PSF, Neat GG (control sample), and GG/3PSF were analyzed by using a high-power benchtop X-ray diffraction (XRD) system manufactured in Karlsruhe, Germany (Bruker D6 Phaser). The testing was conducted at room temperature using a scanning angle range (2θ) between 5° to 80°.

## 4. Results and Discussion

### 4.1. Chemical Analysis by Spectroscopy Technique

**Figure 2:**
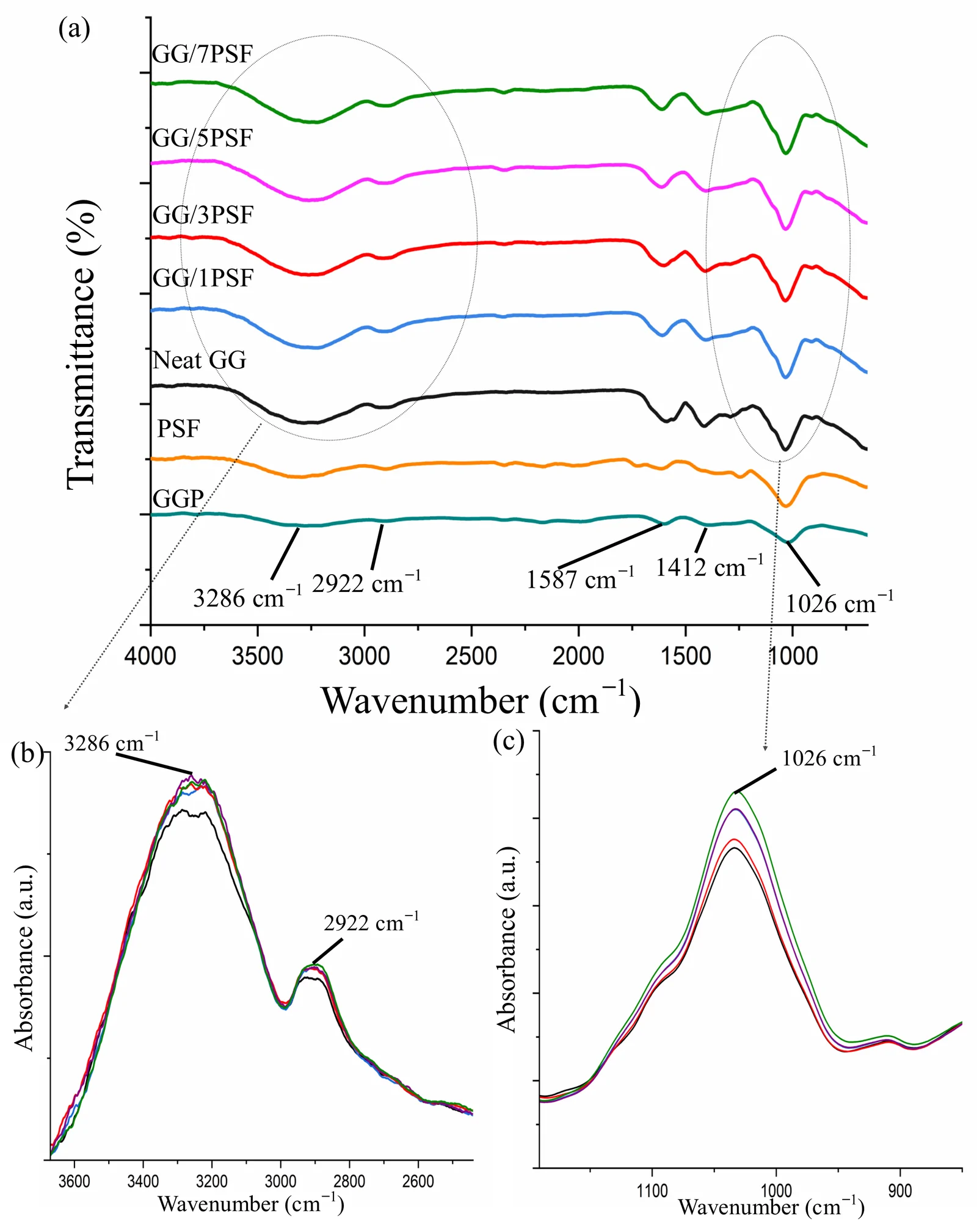
FTIR spectra of GGP, PSF, Neat GG, GG/1PSF, GG/3PSF, GG/5PSF, and GG/7PSF biocomposite buccal films: (**a**) full FTIR spectra; (**b**) enlarged view of the 3286 cm^−1^ and 2922 cm^−1^ regions; (**c**) enlarged view of the 1026 cm^−1^ region.

FTIR spectra of GGP, PSF, Neat GG and GG biocomposite buccal films with different amount of PSF are shown in Figure 2. According to Figure 2a, a broad band at 3286 cm^−1^ indicates the presence of a hydroxyl (-OH) group [7,8,9,10,11,12,13,14,15,16,17,18,19]. The band around 2922 cm^−1^ corresponds to the C-H stretching vibration [19]. The peaks at 1587 cm^−1^ and 1412 cm^−1^ might indicate the vibration of C=O groups that exist in the gellan gum matrix, which has carboxylic acid groups. On the other hand, the presence of a peak at 1026 cm^−1^ was due to the C-O stretching vibration, which is characteristic of the glycosidic bonds and saccharide structure in gellan gum [14]. The PSF is similar to other lignocellulosic fibers, showing a broad peak in the O-H region (3286 cm^−1^) and a strong C-O stretching peak at around 1026 cm^−1^, which is consistent with its cellulosic nature. The FTIR spectrum of the Neat GG film is similar to the GGP with the same main peaks. However, the peaks in the film form (particularly the O-H band and the C-O band at 1026 cm^−1^) are sharper and well-defined in comparison to the powder. This transition is often indicative of a re-organization of the molecular chains and formation of a more integrated network during the film-casting process. The incorporation of PSF caused an alteration in the FTIR pattern, particularly at the peaks around 3286 cm^−1^, 2922 cm^−1^ and 1026 cm^−1^. The band at approximately 3286 cm^−1^ is assigned to O–H stretching vibrations. Its increased intensity upon PSF addition is in line with the alterations in water-associated interactions and the increased contribution of hydroxyl-containing polysaccharide components. However, sample contact, consistency, normalization, and water content might all have an impact on ATR intensity; thus, as a result, the FTIR results do not confirm hydrogen bond formation on their own. The results can be attributed to the rise in hydroxyl groups. PSF contains a high amount of cellulose and other polysaccharides, which have –OH functional groups [20]. Therefore, by adding PSF, the overall number of –OH groups in the film increases, leading to greater absorption at the 3286 cm^−1^ region (refer to Figure 2b). These spectral changes are also consistent with altered hydrophilicity and water interactions. Gellan gum and glycerin both possess water-attracting properties [13]. Consequently, the addition of PSF may boost the moisture content of the film, leading to more O-H interactions, which can also contribute to the increased peak intensity. The absorption peak observed at approximately 2922 cm^−1^ in an FTIR spectrum is typically linked to C-H stretching vibrations, specifically from methylene (–CH_2_) and methyl (–CH_3_) functionalities. This association arises from a higher aliphatic content present in the material. PSF, which is composed of natural cellulose and various polysaccharides, often features extensive carbon chains rich in –CH_2_ and –CH_3_ groups. The incorporation of this filler contributes to an increase in the quantity of these aliphatic groups within the film matrix, resulting in an enhanced absorption peak near 2922 cm^−1^ as can be seen on the samples of GG/1PSF, GG/3PSF, GG/5PSF, and GG/7PSF (refer to Figure 2b). Furthermore, the incorporation of PSF also caused an alteration at the peak around 1026 cm^−1^ (refer to Figure 2c). The prominent peak observed near 1026 cm^−1^ is generally associated with C-O stretching vibrations, a characteristic feature of carbohydrates and polysaccharides. The incorporation of PSF, which is abundant in cellulose and hemicellulose, has the potential to enhance the prevalence of these functional groups within the film matrix, resulting in increased absorbance at this specific wavenumber.

### 4.2. pH Study and Film Thickness

The GG biocomposite oral buccal film pH values and thicknesses are summarized in Table 1. The suitable pH for buccal films typically ranges from 5.5 to 7.5 [21,22]. This range ensures optimal drug solubility, stability, and absorption in the buccal mucosa. The pH measurement provided information on the tolerance of the films, because acidic or basic pH can be irritating to the mucosa. The studied film samples presented values between 6.9 and 7.1, as shown in Table 2. Thus, all the biocomposite buccal film samples were well-tolerated. Film samples with a pH close to neutral (approximately pH 7) are less likely to cause discomfort or irritation when in contact with the buccal mucosa [21,23]. This is an important factor for the samples intended for prolonged contact with oral tissues. The thickness of oral buccal films ranges from 0.05 mm to 0.1 mm [24,25]. Based on Table 2, the film thickness of GG biocomposite oral buccal films was observed to range from 0.06 mm to 0.11 mm. Thinner films can improve patient compliance due to being less noticeable and provide a comforting feeling to them as they are less obstructive in the mouth. The thickness of film samples increases when increasing the PSF content; this is due to the inclusion of more fiber content, which can enhance the viscosity of the mixture, leading to thicker buccal film samples. In this research study, the sequence of thicknesses was GG/7PSF> GG/5PSF> GG/3PSF> GG/1PSF> Neat GG. This was related to the amount of PSF content in each sample.

### 4.3. Tensile Properties of Neat GG, GG/1PSF, GG/3PSF, GG/5PSF, and GG/7PSF Biocomposite Buccal Films

**Figure 3:**
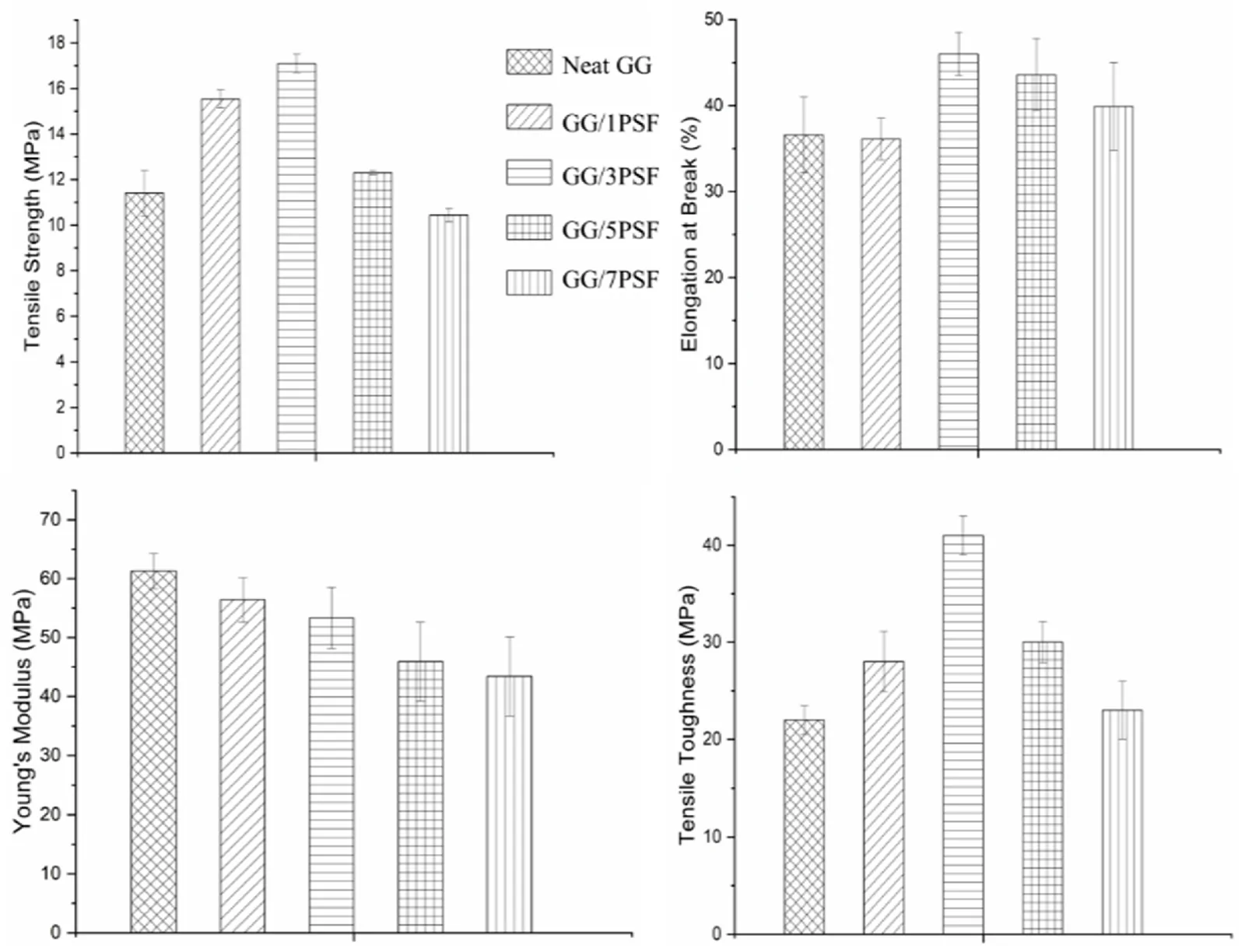
Tensile properties of neat GG, GG/1PSF, GG/3PSF, GG/5PSF, and GG/7PSF of biocomposite films.

The tensile properties such as tensile strength, Young’s modulus, elongation at break and tensile toughness are summarized in Table 3 and clarified in the graph (see Figure 3). As can be seen in Table 3, the addition of PSF was linked to modifications in the GG films’ tensile strength, elongation at break, and tensile toughness, according to the descriptive mean values. These differences are reported as observable numerical changes rather than statistically significant improvements because no inferential statistical analysis was carried out.

Compared to neat GG, the GG/3PSF samples demonstrated a 50% higher tensile strength, alongside enhancements of 26% in elongation at break and 86% in tensile toughness. Several factors may account for the elevated tensile strength, elongation at break, and toughness, alongside a reduced Young’s modulus of the buccal film composed of gellan gum and PSF filler. These fibers can improve the mechanical features of the film by acting as reinforcement, which allows for the material to better endure stress, thus increasing tensile strength [20,26]. The inclusion of these PSFs can enhance the interaction between gellan and the filler, resulting in a composite structure that distributes load more effectively. Tensile strength is defined as the material’s capability to avoid breaking when under tension. The mixture of gellan gum with fibers from the pineapple stem can produce a more robust network that resists deformation, thereby achieving higher tensile strength. Additionally, because the plasticizer boosts flexibility and the fibers offer structural support, the final film can stretch considerably without breaking, leading to a greater elongation at break. Toughness refers to the characteristic of a material that enables it to absorb energy and undergo deformation without breaking. The overall toughness is influenced by a combination of high tensile strength, the flexibility provided by plasticizers, and the reinforcing properties of the fibers. During the mixing phase, fibers may undergo viscous drag as they are distributed within the gellan gum solution. This phenomenon can assist in aligning the fibers with the polymer chains, thereby promoting effective dispersion throughout the matrix. Moreover, both GG and PSF possess hydroxyl groups (-OH) within their molecular structures, as suggested by the FTIR test result (see Figure 2). Although their chemical structures are consistent with potential hydrogen-bonding interactions, the current FTIR data are insufficient to demonstrate the strength or creation of particular hydrogen bonds. It is more reasonable to discuss the enhanced mechanical response as a result of physical interlocking, polymer–fiber compatibility, fiber reinforcement, and potential intermolecular interactions. Depending on the conditions of processing, there are possibilities for cross-linking to take place between PSF and GG molecules. This can increase the strength of the bonds and enhance the mechanical properties. When GG changes from a solution into a gel, the PSF may become trapped within the gel matrix. This trapping helps to stabilize the composite structure and keeps the fibers in the correct position. It is very important that the filler and the matrix work well together. PSFs need to be treated or processed to make sure they spread out evenly in the GG, which can improve the quality of the bonding. The connection between the PSF filler and the GG matrix happens because of mechanical interlocking, hydrogen bonding, and physical trapping during gel formation. This complex relationship results in better properties in the composite material, such as increased tensile strength, flexibility, and toughness. This type of bonding is important for applications like buccal films, where both performance and comfort are very important. High strength and superb flexibility are the two most crucial criteria possessed by the buccal film application. GG biocomposite buccal films show great potential for this application and are a viable alternative to replace other buccal film-based polymers.

### 4.4. Folding Endurance

The folding endurance of a buccal film refers to the film’s ability to withstand repeated bending and folding without breaking or losing its integrity. It is an essential parameter for evaluating the quality and stability of buccal films, which are designed to adhere to the mucosal tissue in the mouth. A higher folding endurance is crucial for patient compliance, ensuring the film remains intact during use, and delivering the intended drug dose. According to Table 4, film samples of Neat GG and GG/1PSF cannot withstand being folded 300 times. Gellan gum, while capable of forming gels, may not possess the mechanical strength required to endure extensive folding without filler (PSF). Its inherent flexibility can lead to breakage under repetitive stress. PSF can reinforce the film matrix because it contains a high amount of cellulose, which possesses strong crystalline structures. These cellulose microfibrils act as reinforcing agents within the gellan gum matrix, improving the tensile strength and stiffness of the biofilm. When dispersed in the polymer network, the fibers help distribute stress more effectively, preventing film breakage. It can improve the tensile strength and stiffness, allowing the film to resist breakage during folding. GG is hydrophilic and can absorb moisture, leading to swelling. This can alter the film’s mechanical properties, making it more susceptible to breakage when flexed repeatedly. GG films can become brittle when dried, making them fragile under mechanical stress. The addition of fibrous fillers can reduce brittleness and enhance flexibility. Additionally, GG molecular structures and interactions may not provide sufficient cohesion under repetitive stress compared to a composite film with added fillers, which can enhance overall strength. Hence, by incorporating PSF or another filler, the composite film can achieve improved mechanical strength, flexibility, and durability, enabling it to withstand a higher number of folds.

### 4.5. Swelling Behaviour of the Buccal Films

The swelling index of oral buccal film typically refers to a measure of how much a film can absorb water and swell when placed in contact with a liquid (buffer solution or simulated saliva) [27,28]. An optimal swelling index in buccal films is influenced by several factors, including the intended drug release profile, the polymer type, and the active pharmaceutical ingredient (API) incorporated [29,30]. However, a moderate swelling index (approximately 100–200%) is generally considered suitable for buccal film because it allows adequate hydration, mucoadhesion, and controlled drug release without causing structural instability [6]. This value refers to the maximum swelling reached within the residence or evaluation time, which in many studies is about 2–8 h, depending on the formulation and intended drug release duration.

**Figure 4:**
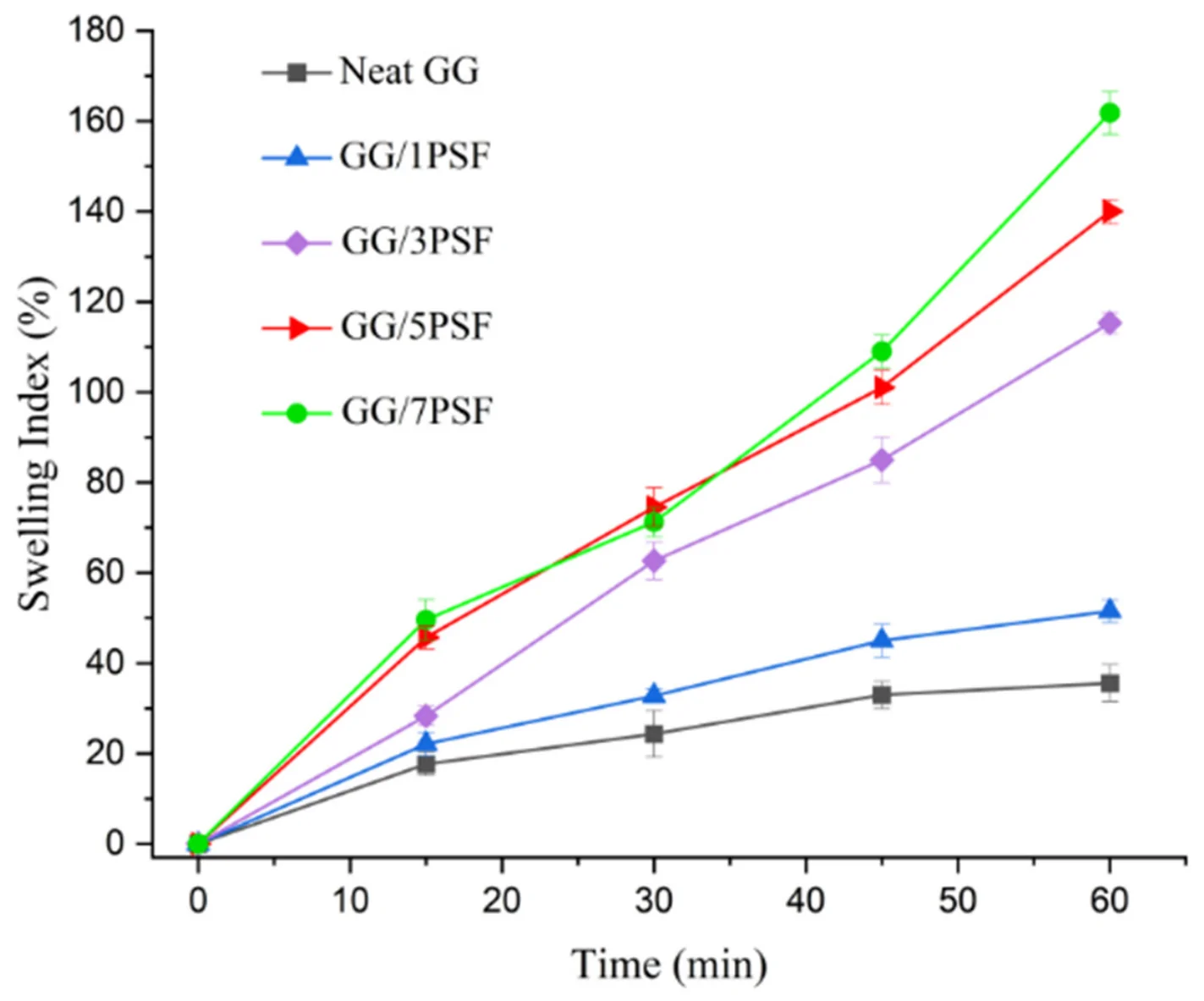
Swelling profiles of Neat GG, GG/1PSF, GG/3PSF, GG/5PSF, and GG/7PSF biocomposite buccal films.

**Figure 5:**
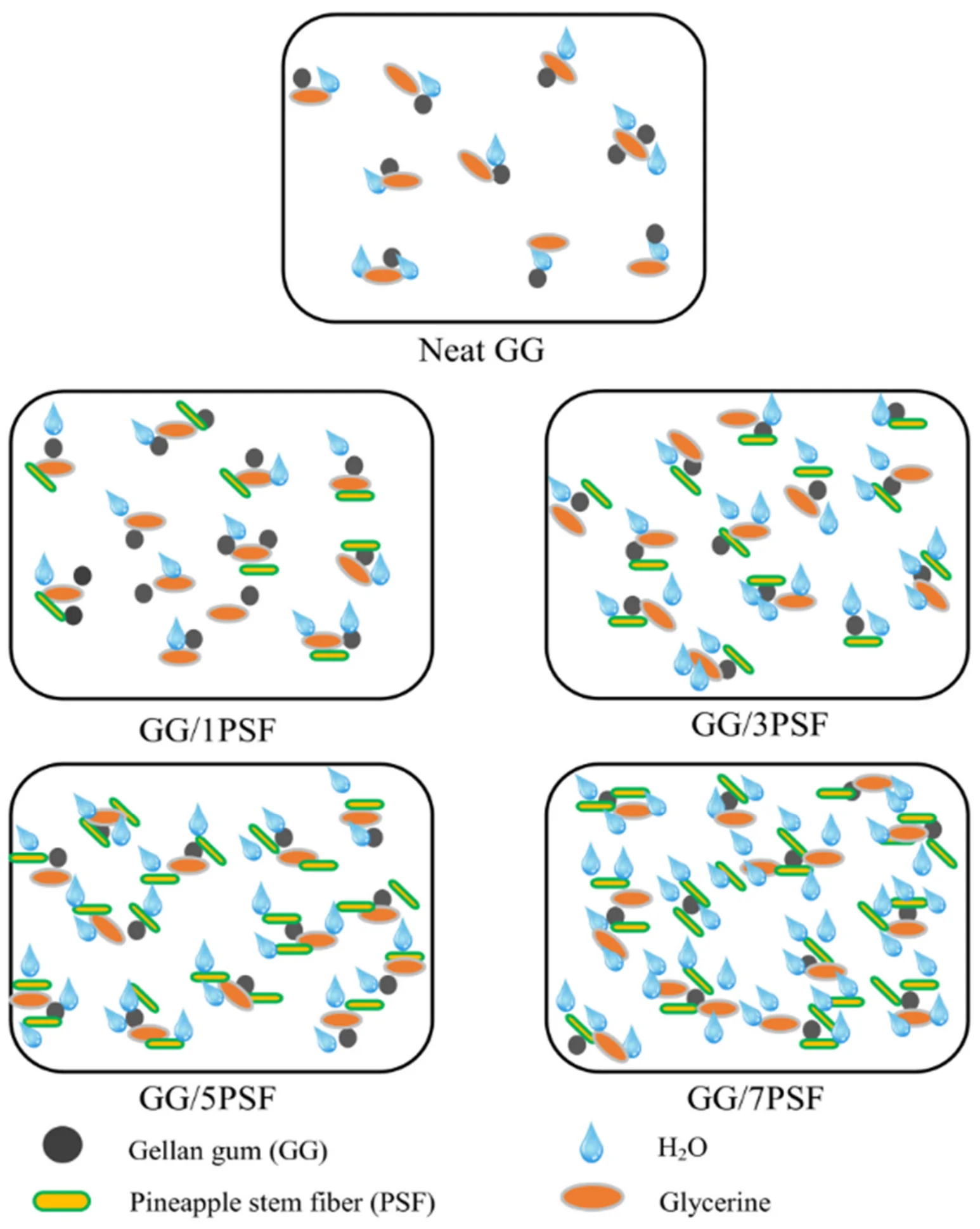
The permeation of water molecules into the Neat GG, GG/1PSF, GG/3PSF, GG/5PSF and GG/7PSF biocomposites oral buccal films. The higher content of PSF allows for the permeation of more water molecules.

The swelling profiles of GG biocomposite buccal films are shown in Figure 4. Biocomposite buccal films of GG/1PSF, GG/3PSF, GG/5PSF, and GG/7PSF that comprised PSF resulted in a higher swelling index after 15, 30, 45, and 60 min of immersing time compared to the Neat GG film sample. The swelling index of the prepared buccal film rates is in the following order: GG/7PSF> GG/5PSF> GG/3PSF> GG/1PSF> Neat GG. The hydrophilic nature of PSF was the reason that the GG/PSF biocomposites exhibited an overall higher swelling index [20]. A hydrophilic fiber (PSF) can absorb and retain moisture, making it suitable for buccal film application due to its potential to enhance drug release. As revealed previously, PSF is hydrophilic; hence, when mixed with GG, which also possesses water-absorbing properties, it can increase the overall water-absorbing capacity of the composite. The FTIR test result appears to confirm this fact (refer to Figure 2). The PSF can generate a network structure within the GG matrix. This network can trap more water, leading to increased swelling. The compatibility of PSF with gellan gum can improve the interaction, allowing the GG to expand more when hydrated. Based on this, an increase in the amount of PSF results in a higher swelling index. The GG/7PSF achieved the highest swelling index among all biocomposite buccal films after 60 min (see Table 5). This phenomenon may be attributed to the higher percentage of PSF, which is 7 wt%, in comparison to other biocomposite buccal film samples, thus indicating an enhanced affinity towards water molecules. Nevertheless, it is important to consider that in the context of oral buccal films, excessive swelling may compromise adhesion or the structural integrity of the film [31]. This assertion is corroborated by the findings from tensile tests (see Table 3), which demonstrate inferior mechanical properties, as evidenced by a notable decrease in tensile strength relative to other samples. As displayed in Table 5, the film samples with PSF had higher water permeation compared to Neat GG (without PSF). Figure 5 illustrates that the higher content of PSF allows for the permeation of more water molecules due to the polar characteristics of the PSF. The PSF might have hydrophilic (water-attracting) properties, which can enhance the interaction between water and GG structures, promoting absorption and permeability. Furthermore, glycerine also provides water-attracting (hygroscopic) properties; when added to GG and PSF, it helps break down the gel structure and facilitates the movement of water within the matrix.

Based on the results, the GG/3PSF film exhibited an optimal swelling index, reaching approximately 115% after 1 h of immersion in simulated saliva fluid (phosphate buffer solution, pH 7.0) under water bath conditions. However, it should be noted that this swelling value may be slightly lower under actual oral conditions, as the volume of saliva in the human oral cavity is considerably smaller than the complete immersion environment used in this experimental setup.

### 4.6. Morphology Analysis

**Figure 6:**
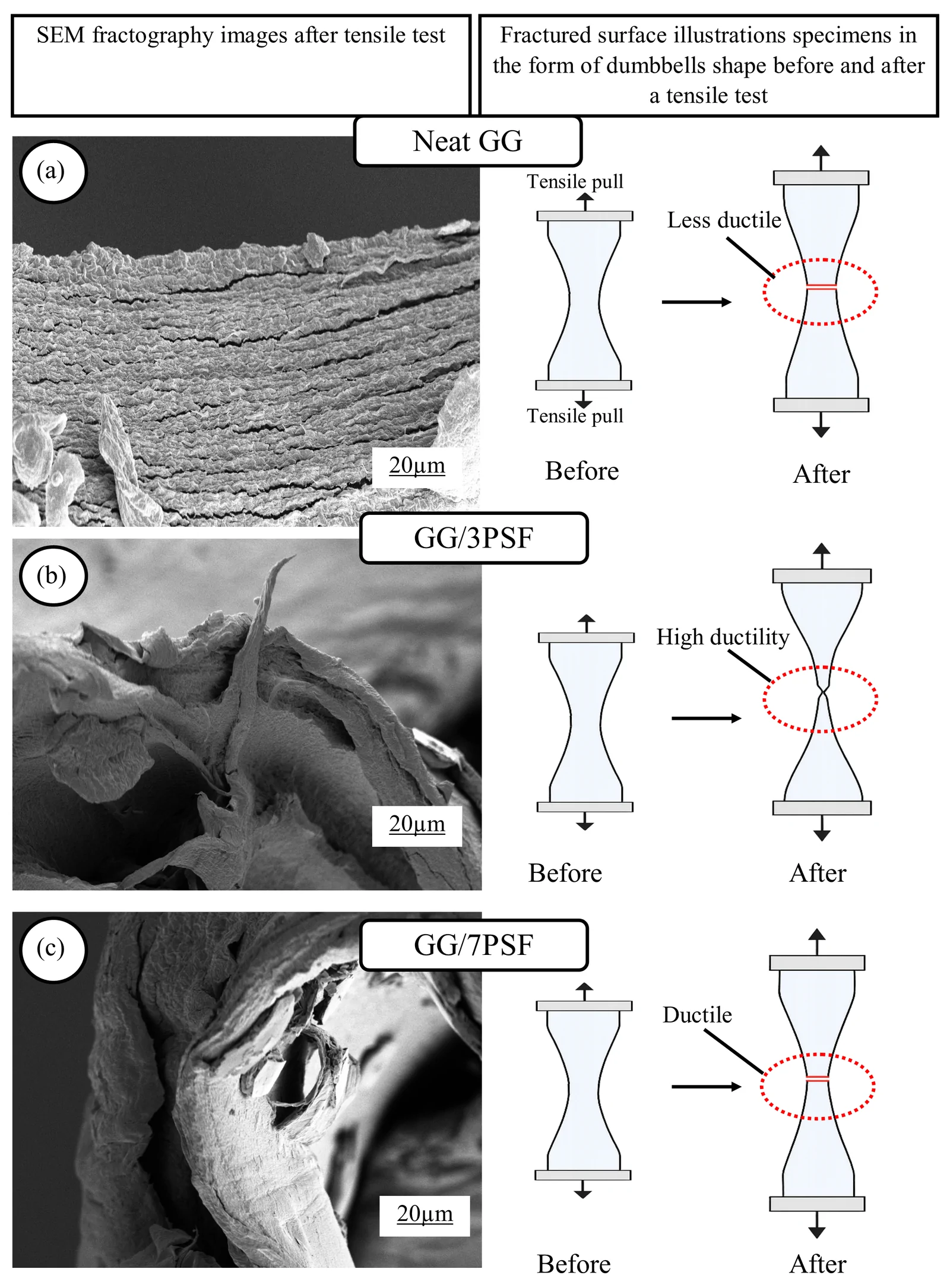
SEM fractography and propagation of fracture across (**a**) neat GG, (**b**) GG/3PSF, and (**c**) GG/7PSF biocomposite film samples.

The SEM micrographs of the tensile fracture surface of the selected sample (Neat GG—control sample; GG/3PSF—optimized sample; GG/7PSF—unoptimized sample) are displayed in Figure 6. Several researchers examined fracture behavior after tensile tests using SEM imaging [32,33].

Based on Figure 6, it is clear that incorporating PSF into the GG matrix results in a more ductile fractured surface compared to pure GG. This enhancement can be attributed to the molecular interactions between GG and the PSF, which contribute to increased ductility. Such interactions appear to form a more cohesive network, allowing the composite to stretch further before breaking, leading to a more flexible film rather than one that snaps. This finding aligns with the tensile test results, which indicate that the elongation at break increases after adding the filler, while Young’s modulus decreases. The inclusion of PSF enhances the elongation at break, allowing the composite to stretch more before it fails. Its fibrous structure enables the material to flex and bend without quickly breaking. This PSF network increases ductility and flexibility [34,35]. Additionally, the addition of PSF might also boost the film’s water retention, which is supported by the swelling test results shown before. Increasing the moisture content can help lessen brittleness, resulting in a more pliable film [36,37]. Essentially, incorporating PSF into a gellan gum matrix forms a composite that merges strength with flexibility, enabling the material to withstand stress while allowing for increased elongation before failure, all while reducing stiffness. This combination of attributes is particularly beneficial for applications such as buccal films, where both comfort (softness) and performance are crucial. In summary, adding PSF to the GG matrix enhances ductility, resulting in a more effective and versatile buccal film.

### 4.7. Mucoadhesion Property of the Buccal Films

The mucoadhesion test is useful for evaluating the residence behavior of oral-mucosal films [38]. This test measures how long the film remains attached to mucosal surfaces and is therefore relevant to oral-mucosal residence behavior. Strong adhesion may increase the duration of material–mucosa contact. However, the effects on drug absorption and bioavailability were not assessed in the present placebo-film study. Additionally, films that stick well tend to improve patient compliance by being more comfortable and allowing for the discreet administration of medication. Although the therapeutic effects were not assessed in this investigation, effective mucoadhesion can lengthen the materials’ residence period on mucosal tissue [39]. The mucoadhesion test is important for the formulation development process as well, guiding choices regarding materials and manufacturing techniques. In this research study, the ex vivo mucoadhesion test was carried out to obtain the results for selected samples (Neat GG, GG/3PSF, and GG/7PSF). It demonstrated how the films were applied to the mucosa surfaces and tested for adhesion strength under specific conditions. The detachment test for assessing bioadhesion from buccal mucosa was a straightforward method used to evaluate the adhesive strength of various formulations. Within the context of this research investigation, fresh goat buccal mucosa was employed because of its close resemblance to human tissue, making it an ideal model for investigating drug absorption and adhesion properties. The choice of goat tissue was also influenced by its availability, particularly in areas where goats are commonly raised for meat, as it tends to be more cost-effective than other animal options.

**Figure 7:**
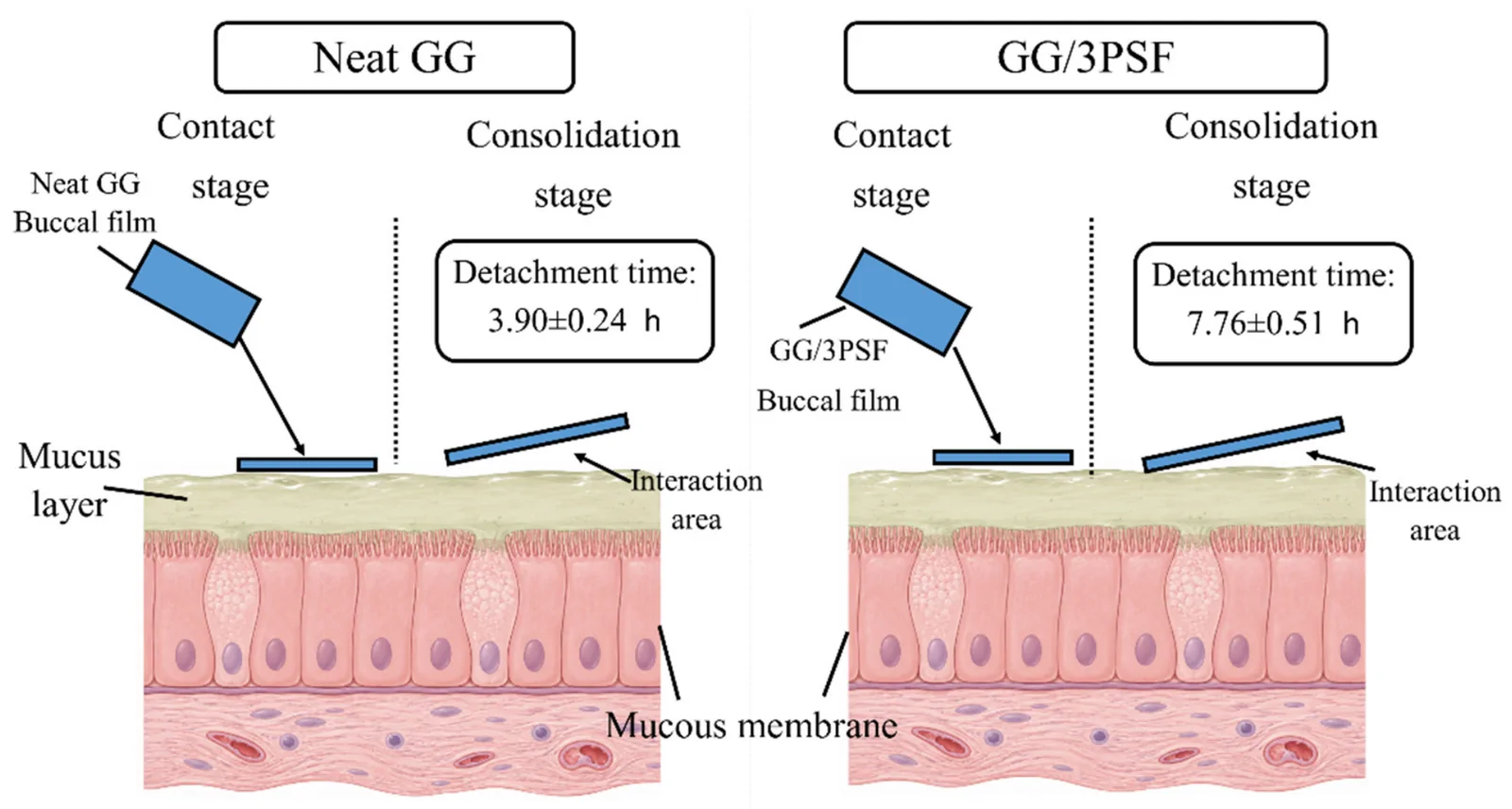
Mechanism of mucoadhesion of Neat GG and GG/3PSF biocomposites buccal films.

The results of the mucoadhesion time for the selected sample are tabulated in Table 6. The findings showed that, in comparison to Neat GG, the samples including PSF with GG in the formulations (GG/3PSF and GG/7PSF) displayed a longer bioadhesion duration. In particular, compared to GG/7PSF (6.48 ± 0.18 h), the GG/3PSF formulation showed the longest mucoadhesion residence time of 7.76 ± 0.51 h. This can be explained by the GG/3PSF’s modest swelling profile (115.31%), which permits the gellan gum chains to interpenetrate with mucosal mucin with adequate hydration. On the other hand, GG/7PSF high fiber content causes overhydration and severe edema (161.81% at 60 min). The residence period is shortened by this over-hydration, which produces a low-viscosity, slick mucilage border layer that experiences cohesive failure and early detachment under the shear stress of the agitation. This provides statistical evidence that GG/3PSF is the best formulation. The residence time within 6 to 8 h is considered optimum for controlled/sustained systemic potential drug delivery applications [40]. Figure 7 shows that the buccal film of Neat GG detached from the mucous membrane at 3.90 ± 0.24 h, while it did so for GG/3PSF at 7.76 ± 0.51 h. An extended mucoadhesion time was observed when the PSF was incorporated into the buccal film formulation (GG/3PSF and GG/7PSF). This can be clarified by several interconnected factors. Primarily, PSF may form a more intricate network with GG, which increases the surface area available for the interaction with the mucosal tissue, resulting in a stronger adhesive bond. Its hydrophilic nature helps in retaining water and swelling when it comes into contact with saliva, thereby improving the contact with the mucosa and enhancing mucoadhesion [41]. Additionally, there may be a positive interaction between GG and PSF, leading to better adhesion to the mucus layer in the mouth. The inclusion of PSF may also change the formulation’s viscosity, which can improve its ability to adhere to mucosal surfaces. Moreover, the texture added by the PSF might create a rougher surface, providing a better grip for the mucus. Lastly, bioactive components in the PSF may interact favorably with the mucosal tissues, further boosting adhesion. Collectively, these aspects contribute to a longer mucoadhesion time, which helps retain the buccal film in the mouth for a prolonged release of its active ingredients.

### 4.8. Crystallinity Analysis 

The aim of carrying out XRD analysis on Neat GG film and GG/3PSF biocomposite film was to explore their structural characteristics. This includes identifying crystalline and amorphous phases, evaluating crystallinity to see how the filler influences the GG polymer structure, and examining the interactions between the GG and PSF.

**Figure 8:**
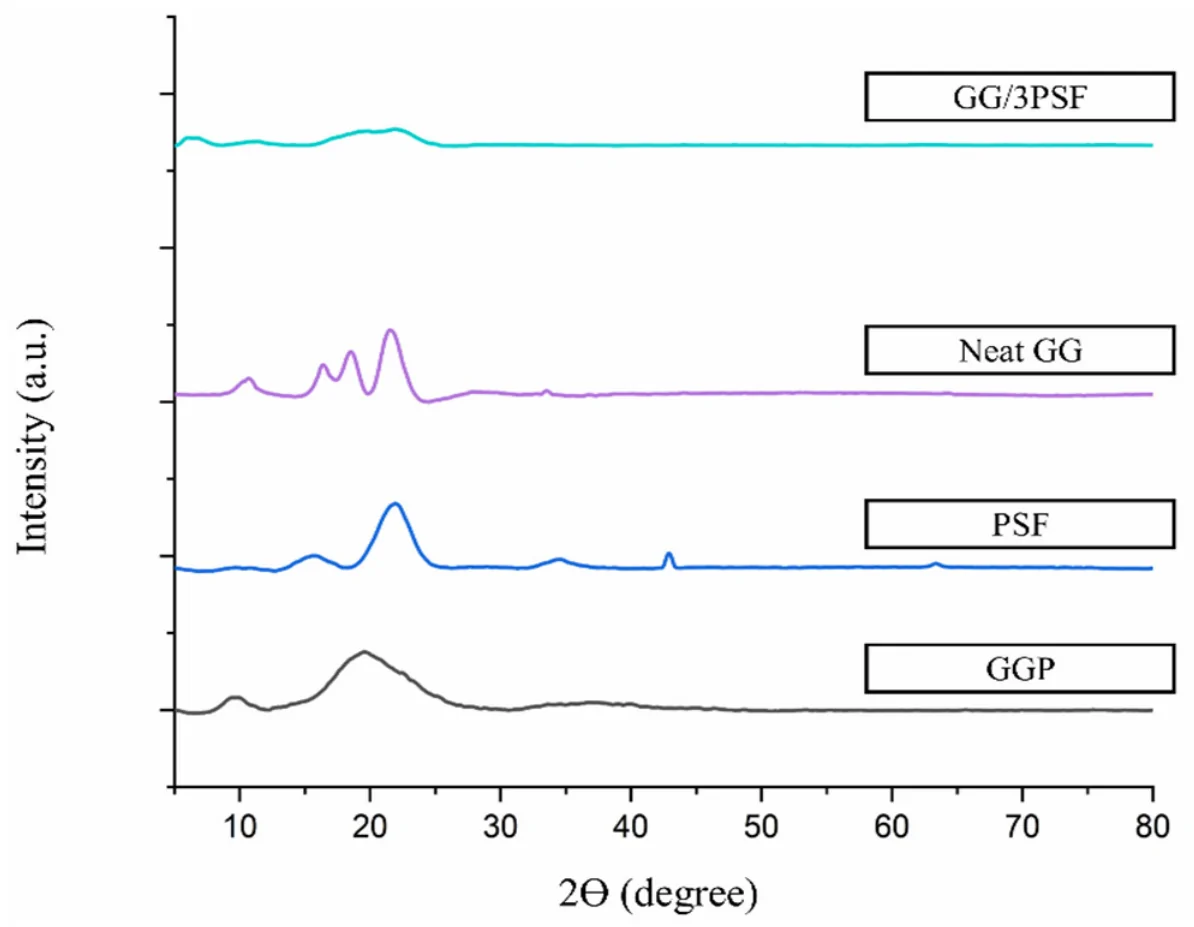
XRD patterns of GGP, PSF, Neat GG film and GG/3PSF biocomposite film.

**Figure 9:**
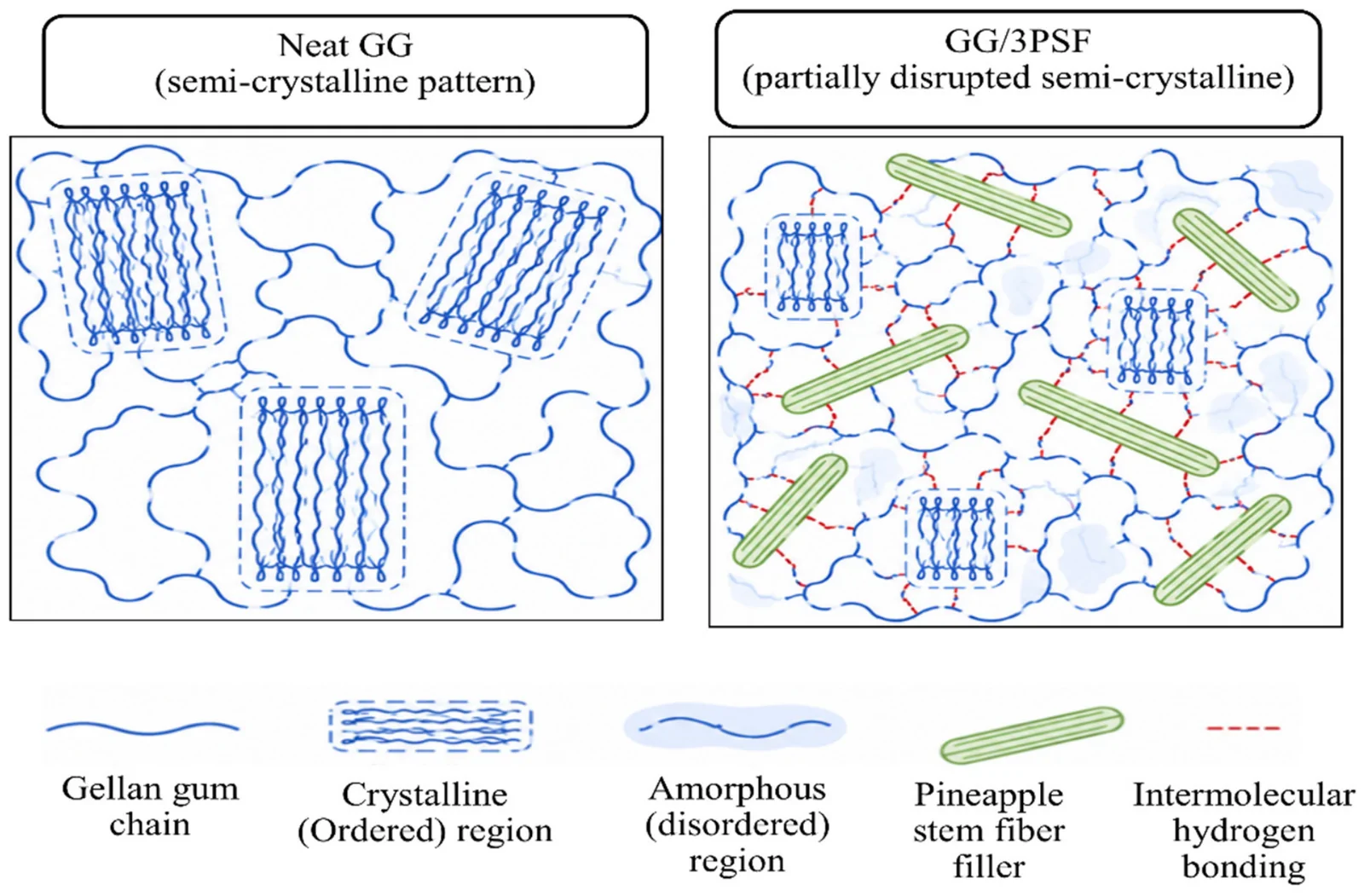
Representation of semi-crystalline (Neat GG film) and partially disrupted semi-crystalline regions (GG/3PSF biocomposite film).

The X-ray diffraction spectra of the GGP, PSF, Neat GG film and GG/3PSF biocomposite film are shown in Figure 8. The GGP shows a semi-crystalline pattern with peaks at approximately 2θ = 9.2° and 19.7°, while the PSF exhibits the typical peaks of cellulose at around 2θ = 15.7° and 21.8°. The material forms a highly ordered semi-crystalline structure when analyzed in a Neat GG film, which is confirmed by sharp diffraction peaks at 2θ = 10.8°, 18.7°, and 21.4°. Nevertheless, there is a noticeable decrease in crystallinity when the PSF is added to the GG matrix, where a wide, diffuse halo in the 2θ range of 19.8° to 21.7° replaces the crisp diffraction peaks of pristine GG. This transition, which is mostly caused by the fiber filler (PSF), shows a disturbance of the regular molecular packing and crystalline organization of the gellan gum chains rather than total amorphization. Strong intermolecular interactions at the fiber–matrix interface probably prevent the GG chains from self-organizing and crystallizing, as shown in Figure 9 [42,43]. The idea that dense molecular interactions serve as a chemical anchor, preventing the gellan gum from organizing into highly ordered crystalline lattices, is supported by the enhanced absorption seen in the O-H stretching region (~3286 cm^−1^) in the FTIR spectra (Figure 2), which is consistent with these interfacial hydrogen-bonding interactions. The observed broadening of the XRD pattern is consistent with the extra hydroxyl-containing components facilitating intermolecular interactions and changing molecular packing. The additional -OH groups enable the formation of large intermolecular hydrogen bonds with the gellan gum and the fiber, disrupting crystallinity. The high frequency of bonding acts as a “chemical anchor” stopping the gellan gum chains from self-organizing into the highly ordered lattice found in the pure gellan gum film. Likewise, the reduction in crystallinity leads to a lower modulus due to the presence of less stiff, ordered regions; however, at the same time, it allows for greater molecular mobility, which accounts for the increase in elongation at break, which is in line with the tensile test results that can be observed in Figure 3 [44].

### 4.9. Study Limitations and Future Perspectives

Although gellan gum and pineapple stem fiber are both well known for being biodegradable, non-toxic, and biocompatible polymers [13,17], the current study did not perform a formal in vitro cytotoxicity screening (such as the MTT assay) on a suitable human cell line (such as gingival fibroblasts or human oral epithelial cells). For any oral-mucosal biomaterial, establishing cell viability and cytocompatibility is an essential precondition. The physical, mechanical, and mucoadhesive characteristics of the created biocomposite are the main topics of this work, which was based on investigating the characterization of basic material. As a result, the next stage of this research, which will come before any clinical translation, will prioritize performing quantitative in vitro cytotoxicity testing and cell viability screening. The current investigation did not directly measure the final films’ moisture contents. As a result, it is impossible to determine how leftover moisture affects the mechanical and swelling characteristics separately. To better understand the connection between film hydration and mechanical behavior, future research should incorporate measures of moisture content or moisture uptake.

## 5. Conclusions

The optimal PSF content plays a crucial role in enhancing the physicochemical properties of the resulting biocomposite film. Based on the several analyses performed, the buccal film formulated with GG and 3 wt% PSF (GG/3PSF) was selected as the optimal formulation. It exhibited a tensile strength of 17.10 ± 0.4 MPa, an elongation at break of 46.0 ± 2.5%, a tensile toughness of 41 ± 2.0 MPa, and an ex vivo mucoadhesive residence time of 7.76 ± 0.51 h, while maintaining a moderate swelling index of 115.31 ± 2.3% (after 60 min), an acceptable neutral pH of 7.0 ± 0.05, and a comfortable thickness of 0.09 ± 0.005 mm. Higher fiber loading (7 wt%) led to excessive swelling (161.81 ± 4.7%) and a reduction in mechanical performance (tensile strength decreased to 10.44 ± 0.3 MPa). These findings indicate that the developed PSF can be effectively utilized as a filler to improve the physicochemical properties of biopolymers, particularly gellan gum. This approach demonstrates significant potential for various oral cavity applications and serves as a promising platform for potential future drug delivery systems, where active pharmaceutical ingredients can be incorporated and tested in subsequent studies. Overall, PSF positively contributes to the performance of buccal films, potentially enhancing both functional properties and patient acceptability.

## Figures and Tables

**Table 1:** Formulation to produce a piece of rectangular-shaped biocomposite buccal film with a size of 14 cm × 11 cm.

Component	Formulation				
	Neat GG	GG/1PSF	GG/3PSF	GG/5PSF	GG/7PSF
**GG (g)**	1	1	1	1	1
**PSF (g)**	0	0.01	0.03	0.05	0.07
**Glycerine (g)**	1	1	1	1	1
**Distilled water (mL)**	50	50	50	50	50

**Table 2:** The range of pH (n = 3, mean ± SD) and film thickness (n = 6, mean ± SD) for the samples, neat GG, GG/1PSF, GG/3PSF, GG/5SF, and GG/7PSF.

Samples	pH Value	Thickness (mm)
**Neat GG**	6.9 ± 0.06	0.06 ± 0.006
**GG/1PSF**	7.0 ± 0.10	0.08 ± 0.006
**GG/3PSF**	7.0 ± 0.05	0.09 ± 0.005
**GG/5PSF**	7.1 ± 0.05	0.09 ± 0.006
**GG/7PSF**	7.0 ± 0.06	0.11 ± 0.006

**Table 3:** Tensile strength, elongation at break, Young’s modulus, and tensile toughness of the neat GG, GG/1PSF, GG/3PSF, GG/5PSF and GG/7PSF biocomposite films (n = 5, mean ± SD).

Type of Samples	Tensile Strength (MPa)	Elongation at Break (%)	Young’s Modulus (MPa)	Tensile Toughness (MPa)
**Neat GG**	11.40 ± 1.0	36.6 ± 4.4	61.3 ± 3.0	22 ± 1.5
**GG/1PSF**	15.54 ± 0.4	36.1 ± 2.4	56.4 ± 3.8	28 ± 3.1
**GG/3PSF**	17.10 ± 0.4	46.0 ± 2.5	53.3 ± 5.2	41 ± 2.0
**GG/5PSF**	12.30 ± 0.1	43.6 ± 4.2	45.9 ± 6.7	30 ± 2.1
**GG/7PSF**	10.44 ± 0.3	39.9 ± 5.1	43.4 ± 6.7	23 ± 3.0

**Table 4:** Folding endurance results of Neat GG, GG/1PSF, GG/3PSF, GG/5PSF, and GG/7PSF.

Samples	Folding Endurance
**Neat GG**	<300
**GG/1PSF**	<300
**GG/3PSF**	>300
**GG/5PSF**	>300
**GG/7PSF**	>300

**Table 5:** Swelling test results of biocomposite buccal films (Neat GG, GG/1PSF, GG/3PSF, GG/5PSF, and GG/7PSF (n = 3, mean ± SD).

Time (min)	Samples and Swelling Index (%)				
	Neat GG	GG/1PSF	GG/3PSF	GG/5PSF	GG/7PSF
**15**	17.67 ± 2.5	22.10 ± 2.7	28.33 ± 2.1	45.67 ± 2.6	49.63 ± 4.5
**30**	24.33 ± 5.1	32.77 ± 1.5	62.65 ± 4.1	74.53 ± 4.3	71.25 ± 3.2
**45**	33.00 ± 3.0	45.00 ± 3.6	85.00 ± 5.0	101.02 ± 3.8	109.00 ± 3.6
**60**	35.56 ± 4.0	51.55 ± 2.5	115.31 ± 2.3	140.00 ± 2.7	161.81 ± 4.7

**Table 6:** Mucoadhesive residence time of buccal films (Neat GG, GG/3PSF, and GG/7PSF) (n = 3, mean ± SD).

Samples	Detachment Time (h)
**Neat GG**	3.90 ± 0.24
**GG/3PSF**	7.76 ± 0.51
**GG/7PSF**	6.48 ± 0.18

## Data Availability

The original contributions presented in the study are included in the article; further inquiries can be directed to the corresponding author.
